# Determination of the Initial Abnormal Discharge Site in Temporal Lobe Epilepsy Through Combined EEG and Neuroimaging. What Is Next?

**DOI:** 10.3389/fneur.2021.798228

**Published:** 2021-12-24

**Authors:** Ana A. Rentería-Palomo, Jose L. Montes-Ochoa, Adriana Martinez-Mayorga, Jorge Guillermo Reyes-Vaca, Ildefonso Rodríguez-Leyva

**Affiliations:** Facultad de Medicina, Neurology Service, Hospital Central Dr. Ignacio Morones Prieto, Universidad Autónoma de San Luis Potosí (UASLP), San Luis Potosí, Mexico

**Keywords:** TLE, MRI, EEG, hippocampal volume, epileptic seizure

## Abstract

**Objective:** The objective of this study was to determine the relationship between atrophy of the hippocampus and severity of epilepsy in patients with temporal lobe epilepsy (TLE) as the first step to evaluate the possibility of surgery for epilepsy and analyze why patients cannot undergo epilepsy surgery.

**Methods:** Volumetric MRI of the hippocampus was performed in 51 consecutive patients (29 men; mean age 40) with TLE. TLE diagnosis, lateralization, and severity (mild, moderate, severe) of seizures were based on a comprehensive evaluation that included neurologic examination and EEG in all patients. Patients with evidence of a lesion other than hippocampal sclerosis were not included in the study. We assessed the relationship between hippocampal volumes and electrophysiological evidence of seizure severity.

**Results:** According to the affected side based on the EEG, a statistically significant difference (*p* < 0.001) in volume and a positive correlation between epilepsy and hippocampal atrophy were found.

**Conclusion:** Our results confirm that volume loss to the hippocampus in patients with TLE correlates with the severity of epilepsy based on the EEG. Therefore, surgical treatment is considered early when hippocampal atrophy is evident in patients with refractory TLE. However, in Latin American countries, it is a challenge to get a patient to undergo epilepsy surgery. Therefore, we try to analyze the sad situation in our hospital.

## Introduction

Epilepsy [according to the International League Against Epilepsy, ILAE, and the International Bureau for Epilepsy, IBE, (2005)] is defined as a brain disorder characterized by predisposition to generate recurrent epileptic seizures, which usually leaves neurobiological, cognitive, psychological, and social consequences, in which the presence of at least one epileptic seizure is necessary (1). A task force of the ILAE proposed that epilepsy is a brain disease defined by (1) at least two unprovoked (or reflex) seizures within more than 1 day; (2) one unprovoked (or reflex) seizure and probability of new seizures similar to the overall risk of recurrence (at least 60%) after two unprovoked seizures, occurring within the next 10 years; (3) diagnosis of an epileptic syndrome (2).

An epileptic seizure is a recurrent event characterized by the transient presence of signs and symptoms due to abnormal overactivity or synchronous neuronal activity in the brain (1).

Factors that influence the form of manifestation of an epileptic seizure are: (a) the location of the abnormal neuronal overactivity in the brain, (b) the mode of propagation, (c) brain maturity, (d) the patient's underlying disease, (e) the sleep-wake cycle, (f) medications, and (g) the clinical semiology of seizures, among others. Besides, the EEG and MRI (3) can help localize the abnormality in function or structure of the brain, especially in temporal lobe epilepsy (TLE).

In 1989, TLE was included in the ILAE classification under the group of symptomatic localization-related epilepsies. The definition offers a tentative description based on suggestive clinical features plus ictal and interictal electroencephalogram (EEG) findings. The ILAE also identifies seizures originating from the amygdalohippocampal area (mesio-basal limbic or rhinencephalic) and the lateral temporal area. The other form of TLE is often referred to as neocortical TLE (nTLE) (4). In the new classification of seizures, most TLE seizures are recognized as focal, with impaired awareness, motor onset and automatism, non-motor onset and behavioral arrest, or cognitive alteration sometimes with bilateral tonic-clonic propagation (5).

Seizures can affect sensory, motor, and autonomic functions, and consciousness, emotions, cognitive, memory, and behavior. Not all seizures affect these sensory, motor, and autonomic functions; however, at least one is involved. Sensory manifestations include somatosensory, auditory, visual, olfactory, gustatory alterations, and more complex presentations, such as perceptual distortions (6).

Cognitive disturbances may appear as problems in perception, attention, emotion, memory, performance, or language. The emotional state is difficult to define in a similar way for patients. Still, it must be considered, since some cases present with manifestations of fear, anxiety, satisfaction, and joy. Hemispheric lateralization of TLE is not often straightforward, and abnormal activity may rapidly propagate to the contralateral hemisphere to other cerebral presentations of clinical symptoms related to secondary sites. Careful observation of ictal semiology, however, can help identify the lateralization in TLE. To localize the seizure onset zone in TLE, we can utilize:

1. unilateral clonic activity (with contralateral focus),
2. unilateral dystonic or tonic posturing (in 90 and 86%, respectively),
3. unilateral automatisms (with ipsilateral seizure focus in 80%),
4. ictal speech preservation (with seizure focus contralateral to the dominant language hemisphere in 80%), the preservation of language, is not localizing; it just informs whether the seizure impacts or not the language areas, and
5. versive head rotation occurring ≤10 s before seizures consistently generalized predicted a contralateral focus.

Less predictive manifestations of lateralization included ictal speech arrest and postictal speech status, with predictive values of 67%. Eye deviation, aura type, and versive head movements occurring at times other than secondary seizure generalization were less predictive of lateralization (7). Sometimes, it is possible to recognize the lateralization but not the affected lobe; several differences can be that frontal lobe seizures are preceded by somatosensory auras localized to the chest or epigastrium olfactory auras suggesting orbitofrontal lobe involvement. Epigastric auras commonly precede TLE seizures. Parietal lobe seizures present with somatosensory aura, numbness, pain, and tingling sensations. Visual auras commonly precede occipital lobe seizures (8).

Temporal lobe epilepsy usually presents with focal seizures. However, the underlying pathology can be any of a wide range of conditions, such as hippocampal sclerosis, low-grade glial tumors (disembryoplastic neuroepithelial tumor, ganglioglioma, and oligodendroglioma), migratory neuronal disorders (cortical dysplasia), and vascular lesions (cavernous malformation and arteriovenous malformation), although in a significant number of cases no structural abnormalities are found (9). At least one-third of patients with TLE develop medically intractable epilepsy (IE), which is a common form of epilepsy; it is necessary to identify pharmacological, genetic, neurobiological, and immunological factors to improve the prognosis of those suffering from this problem (10). Therefore, there has been a growing interest in surgical therapy for medically IEs. Cortical resection of the seizure onset zone is the most widely accepted model of surgical management. Still, anatomically standard resection of the temporal lobe or resection limited to amygdalohippocampectomy can be a possible route to improve the quality of life in subjects with IE of temporal lobe origin. Especially, if the procedure is made early, the approach provides optimum balance of benefits to risks and costs for all patients with TLE (11).

A study on patients with IE, searching for structural changes and their localization in the brain, could be relevant for understanding the expression of epileptic seizures. Particularly, MRI-based volumetric measurements of the amygdala and hippocampus have been proven to be helpful in the diagnosis and treatment of patients with TLE. This imaging modality correlates amygdala and hippocampal volumes with semiological, neurophysiological, and neuropathological findings, post-surgical outcome, and clinical course. Technical and anatomical aspects underlying the successful use of this modality that has been reported in previous studies were evaluated. However, the sensitivity of qualitative visual analysis vs. quantitative volumetric MRI analysis is a matter of debate. When used in conjunction with electroencephalographic monitoring, volumetric MRI will allow us to treat patients with TLE appropriately, efficiently, and cost-effectively, giving us the ability to offer surgery earlier, especially in developing countries (12).

## Materials and Methods

This study was performed at the Central Hospital “Dr. Ignacio Morones Prieto” in San Luis Potosi, Mexico. Fifty-one consecutive patients with idiopathic TLE (22 women and 29 men, with an average age of 40 years)1 were recruited between 2010 and 2011.

The 51 patients selected met the following inclusion criteria: both genders, >18 and <80 years, with an average age of 44 years old, diagnosis of idiopathic temporal lobe IE, EEG, and MRI. Exclusion criteria were neuroimaging showing tumors, infections, or infarcts.

A neurologist with electrophysiological training analyzed the electroencephalographic studies to classify the right and left temporal lobe epilepsy groups. In turn into three subgroups with categories of mild, moderate, or severe, according to the EEG characteristic's findings (according to the frequency of the presence of epileptic graphoelements in the EEG, obtained an average per time; mild: more than 1 per h but less than 1 per min; moderate: more than 1 per min but <1 in 10 s; severe: more than 1 per s) by a neurophysiologist who was blind to the semiology of the seizures For the imaging studies, the Signa 1.5 T GE superconductive high-field magnetic resonance imaging equipment was used, with an 8-channel neurovascular coil and T1- and T2- weighted images.

Hippocampal volumes were derived using Analyze 10.0 (Mayo Clinic) by manually delineating the structure on the T1-weighted images. We used as control the contralateral hippocampus that was not damaged or was less affected in each participant.

## Results

Measurements were made for each patient in both hippocampal areas. Then, the results were compared, subtracting the left hippocampal area from the right. Therefore, positive values would indicate a larger size in the right hippocampal region, and negative values would show a larger space in the left hippocampal area.

Ten years after completing this research, we have not implemented epilepsy surgery routinely at our institution.

**Figure 1:**
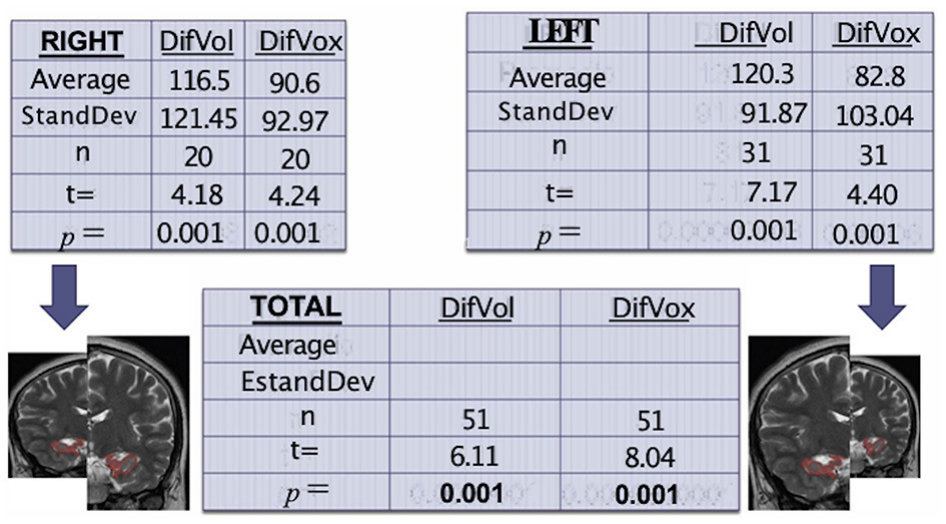
Tables show the correlation among volume, voxels, and hippocampal atrophy, comparing the affected side against its control (itself). Again, the results show a high sadistic significance.

These findings are exemplified in Figure 1.

**Figure 2:**
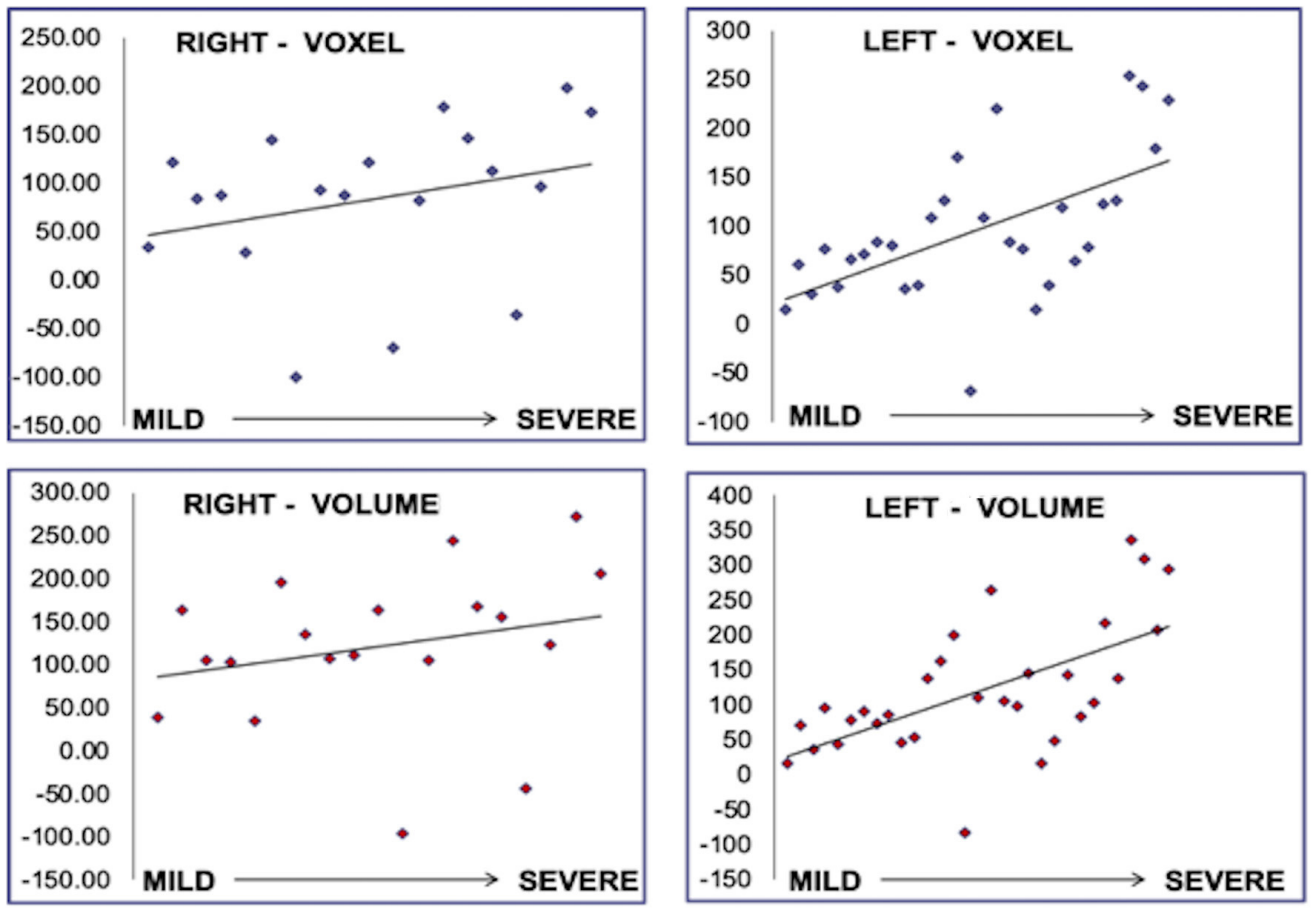
Correlation between voxel and volume with severity of the findings in the EEG. The results are presented for each IE group: mild, moderate, and severe, and each affected side, left and right.

Despite being blinded, the neurophysiologist achieved an interpretation associated with the location and greater involvement in the volumetric and voxel measurements of the hippocampal area (Figure 2).

## Discussion

We used methods such as EEG and mathematical analysis of MRI based on four variables (area, volume, voxels, and intensity) correlated more strongly than the usual qualitative methods for MRI assessment.

We found that the mathematical measures that correlated most strongly with EEG were volume and voxels, showing statistical significance in all six classified groups. Thus, we can detect a very high correlation between EEG and MRI, precisely the quantitative measures of volume and voxels based on these two measures. We also found a correlation between the severity of epilepsy and the more significant difference between hippocampi concerning volume, area, and voxels. Unfortunately, the next step is very challenging to establish. The reason is that we are cautious in several essential points: (a) lack in the number of specialists to perform this kind of surgery, (b) lack of adequate equipment to make the location of the epileptic focus more precise, even if we already know the side and the size (e.g., applying recording racks, deep electrodes), (c) belonging to public institutions where a patient who attends does not have financial resources and the institution lacks the means to support his preoperative study and performance of the procedure.

## Conclusions

Magnetic resonance imaging (MRI) has revolutionized the detection of structural abnormalities in patients with epilepsy. However, many focal abnormalities remain undetectable to routine visual inspection by the observer. MRI analysis using specialized software optimizes the parameters and may provide a tool for further clinical evaluation. Improve detection previously occult malformations; thus, improving the identification of patients who may benefit from epilepsy surgery by more accurately detecting the irritative focus. Matching semiology of the seizures, EEG, and MRI image localization provide us with a valuable armamentarium to improve the prognosis of IE in patients with TLE (13–16). The lack of human and economic resources to complete the diagnostic approach and perform an early surgical procedure favors that our patients continue suffering from refractory epilepsy and have a higher risk of premature death (SUDEP).

## Author Contributions

AR-P was the investigator who collected the data, clinical history, EEG and imaging studies of the patients, and also analyzed the electroencephalograms and classified them. AM-M was the neurologist and neurophysiologist in this study. JR-V a neuroradiologist, reviewed the images and supervised the analysis of data. JM-O reviewed the results, shared ideas, and edited the article. IR-L was the principal investigator, who coordinated the study, drafted the article, and organized the participation of all the authors. All authors contributed to the article and approved the submitted version.

## Conflict of Interest

The authors declare that the research was conducted in the absence of any commercial or financial relationships that could be construed as a potential conflict of interest.

## Publisher's Note

All claims expressed in this article are solely those of the authors and do not necessarily represent those of their affiliated organizations, or those of the publisher, the editors and the reviewers. Any product that may be evaluated in this article, or claim that may be made by its manufacturer, is not guaranteed or endorsed by the publisher.
